# Inhibitory Effect of *Lactiplantibacillus* *plantarum* and *Lactococcus lactis* Autochtonous Strains against *Listeria monocytogenes* in a Laboratory Cheese Model

**DOI:** 10.3390/foods11050715

**Published:** 2022-02-28

**Authors:** Maria Barbara Pisano, Maria Elisabetta Fadda, Silvia Viale, Maura Deplano, Federica Mereu, Marijana Blažić, Sofia Cosentino

**Affiliations:** 1 Department of Medical Sciences and Public Health, University of Cagliari, Cittadella Universitaria, SS 554, Km 4,500, 09042 Monserrato, Italy; barbara.pisano@unica.it (M.B.P.); mefadda@unica.it (M.E.F.); silviaviale@virgilio.it (S.V.); mdeplano@unica.it (M.D.); federica.mereu94@unica.it (F.M.); 2 Department of Food Technology, Karlovac University of Applied Sciences, Trg J.J.Strossmayera 9, 47000 Karlovac, Croatia; marijana.blazic@vuka.hr

**Keywords:** antimicrobial, lactic acid bacteria (LAB), bacteriocin, biopreservation, *Listeria monocytogenes*

## Abstract

In the present study, six *Lactococcus lactis* and seven *Lactiplantibacillus plantarum* strains isolated from artisanal Sardinian dairy products were evaluated for their efficacy in controlling the growth of *Listeria monocytogenes* during the storage of miniature fresh cheese manufactured on a laboratory scale to exploit their possible use as biopreservatives. The strains were tested for antimicrobial activity and some technological characteristics before using them in miniature fresh cheese to evaluate their in situ antilisterial effect. Our results showed that five strains (*L. lactis* 16FS16-9/20234-11FS16 and *Lpb. plantarum* 1/14537-4A/20045) could be considered suitable candidates for use as protective cultures in fresh cheese manufacture since they significantly lowered the pathogen counts by 3–4 log units compared to the control; however, all strains tested were capable of decreasing *L. monocytogenes* numbers. Our results suggest that the single and combined action of the acidifying power and the production of bacteriocin of these strains was capable of controlling and/or reducing the growth of *L. monocytogenes*. Considering their technological characteristics, they might be used as starter/adjunct cultures to increase the safety of the products, perhaps in association with other antimicrobial hurdles.

## 1. Introduction

The continuous attention of consumers versus natural and healthy food has led food research and industry to investigate the use of naturally occurring compounds (biopreservatives) in foods processing and preservation and to reduce the utilization of chemical additives as antimicrobials [1]. To this end, the use of lactic acid bacteria (LAB) has been described as an interesting approach for food biopreservation in alternative to chemicals and is also regarded by the consumer as a lower risk food preservation [2,3].

LAB are widespread, occur naturally in many plants and animal food sources and have been safely used for a long time in the production of dairy and other fermented products, as attested by the attribution of QPS (Qualified Presumption of Safety, in EU) and GRAS (Generally Recognized as Safe, in the USA) status [4,5,6,7]. Today, LAB strains are widely used as starter/adjunct cultures in food production and provide the benefits of microbiological safety, uniformity, quality and durability in the final product [8,9,10,11]. In addition to heightening the technological and organoleptic properties of the final products, some strains have the ability to control the development of undesired or pathogenic microorganisms such as *Listeria monocytogenes*, *Bacillus cereus*, *Staphylococcus aureus* and *Clostridium tyrobutyricum* [12] and further improve food quality, safety and shelf-life [9]. In fact, the production of pathogen-inhibiting substances such as organic acids, hydrogen peroxide, CO2, diacetyl or antimicrobial peptides, i.e., bacteriocins, is a well-recognized ability in LAB [13,14,15]. These features have been demonstrated to be strain-dependent; therefore, studies reporting the screening of LAB of different origins with the purpose of discovering new probiotic or starter strains are rising by the day [8,16].

Among LAB, the species *Lactiplantibacillus plantarum* (formerly known as *Lactobacillus plantarum*) and *Lactococcus lactis* are known to be naturally present in a variety of fermented food products, where they have been safely used for a long time [17,18].

*Lpb. plantarum* is considered one of the most important species of lactobacilli because of its technological and probiotic characteristics. Various *Lpb. plantarum* strains have been demonstrated to produce several antimicrobial agents against pathogenic or spoilage microorganisms. This antimicrobial impact has frequently been linked to the production of organic acids, such as lactic and phenyllactic acids [19,20,21].

*Lactococcus lactis* strains are the main LAB components of starter cultures used for the production of many fermented dairy products [22]. In cheese manufacture, the major characteristic of lactococci strains is their ability to quickly acidify milk, thus also contributing to the development of higher sensory properties (i.e., taste, flavor and texture) in cheeses [23]. Moreover, the occurrence of *Lactococcus* in cheeses has been linked with the increased microbial safety and stability of the products during storage, related to the production of substances (e.g., organic acids, bacteriocins, fat and amino-acid metabolites) capable of inhibiting spoilage and pathogenic microorganisms [13,24].

Foodborne diseases transmitted by dairy products still represent a significant safety issue in both developed and developing countries. Contamination can occur at any point in the supply chain and may arise from several sources (e.g., environmental, animal and human) [25]. It is therefore mandatory to control pathogens not only during milk production but also throughout cheese manufacturing. Cheese is, in fact, an efficient vehicle for foodborne pathogens, particularly fresh cheeses manufactured from raw milk. However, this contamination can also be present in cheese produced with pasteurized milk as a consequence of post-pasteurization contamination [26]. Due to the high pH and water activity combined with conservation at 4 °C, fresh cheese warrants special consideration from a hygienic/safety point of view [27]. The microbial contamination of these products is crucial for the dairy industry due to economic losses and for public health because of the risk of transmitting pathogenic microorganisms, such as *Listeria monocytogenes*, to consumers. The major concern related to the occurrence of *L. monocytogenes* in foods is the possibility of developing listeriosis, one of the most severe foodborne diseases in developed countries [28]. Some characteristics of *L. monocytogenes*, such as its broad occurrence and ability to tolerate environmental stresses, including low pH, low temperatures and salt concentration up to 10% [29,30], make it difficult to contain its presence in dairy foods. As a result, contaminated cheese has been involved in some of the major listeriosis outbreaks recorded in the world [27,31,32,33]. Soft-fresh cheeses are particularly vulnerable to contamination by *L. monocytogenes* because the processing and consumption of these products allow suitable conditions for its growth [13,34,35]. Controlling *L. monocytogenes* is crucial to food safety considering listeriosis’ high mortality rate, especially among susceptible populations, such as pregnant women, newborns, the elderly and those with compromised immune systems [36].

The antilisterial activity of *L. lactis* strains is generally related to their ability to produce bacteriocins. In a previous study, we showed that six bacteriocin-producing *L. lactis* subsp. *lactis* strains isolated from artisanal Sardinian dairy products were able to inhibit *L. monocytogenes* during cocultivation in skimmed milk, decreasing the counts of approximately 4 log units compared to the positive control after 24 h of incubation [37].

Antibacterial activity, supposedly due to organic acid production by *Lpb. plantarum* isolated from dairy products, has also been reported [3,38,39,40]. Recently, we have shown that the production of high levels of organic acids positively correlated with the antifungal activity in *Lpb. plantarum* strains [41].

In the present study, *L. lactis* and *Lpb. plantarum* strains isolated from artisanal Sardinian dairy products were evaluated for their efficacy in controlling the growth of *L. monocytogenes* during the storage of miniature fresh cheese manufactured at laboratory scale.

## 2. Materials and Methods

### 2.1. Bacterial Strains and Culture Conditions

Six *Lactococcus lactis* and 7 *Lactiplantibacillus plantarum* strains isolated from raw milk and artisanal ewe’s cheeses were tested in the study. The strains were identified by phenotypic tests and genetic analysis based on polymerase chain reaction amplification using species-specific primers derived from 16S rRNA sequences, as previously reported [42] and are deposited in the MicroBioDiverSar (MBDS) culture collection (www.mbds.it, accessed on 18/01/2022). The strains were stored at −20 °C in MRS broth (Microbiol, Cagliari, Italy) with 15% (*w*/*v*) glycerol and propagated three times in MRS broth for activation before experimental use. *Listeria monocytogenes* ATCC 7644, *Escherichia coli* ATCC 25922 and *Staphylococcus aureus* ATCC 25923, used as indicator strains in the antimicrobial activity assay, were stored on nutrient broth (NB, Microbiol) plus 20% (*w*/*v*) glycerol at −20 °C and subcultured twice in the appropriate medium before use.

Of the 13 strains tested, 4 lactococci were characterized in previous studies as producers of antimicrobial substances and were also able to inhibit *L. monocytogenes* during cocultivation in skimmed milk [37,43,44,45], and 2 lactobacilli were shown to produce in vitro high levels of organic acids, particularly phenyllactic acid [41]. The remaining strains were included in this study because of their antimicrobial and technological features [3,39,46,47]. All strains were able to grow at 10 °C (data not shown). The main characteristics of the strains used in this study have been outlined in Supplementary Table S1.

### 2.2. Antimicrobial Activity Assay

The LAB strains were screened for antimicrobial activity against the indicator strains reported above using the agar spot test method as described by Schillinger and Lücke [48]. Briefly, 3 μL aliquots of a fresh overnight culture of the strains were spotted onto the surface of MRS agar (1.2% (*w*/*v*) agar-0.2% (*w*/*v*) glucose) plates. After incubation in a GasPak anaerobic jar (GENbox anaer, BioMeriéux, Marcy L’Etoile, France) for 24 h at 30 °C, the plates were overlaid with 7 mL soft agar medium (NB containing 0.7% *w*/*v* agar) seeded with the indicator strain to a final concentration of approximately 10^7^ colony-forming unit (cfu)/mL. After 24 h of incubation at the optimal growth temperature and atmosphere for the indicator strains, the presence of a detectable clear zone around the colony of the producer strain, which was considered indicative of inhibition, was measured.

### 2.3. Technological Characteristics

The caseinolytic activity was analyzed in plate count agar (PCA) (Microbiol) with 10% sterile reconstituted skim milk (RSM, Oxoid, Milan, Italy). After incubation at 30 °C in aerobiosis, the presence of clear zones around the colony was verified. The lipolytic activity was determined on MRS (Microbiol) with 0.1% tributyrin. Lipolytic colonies were surrounded by clear zones against a turbid background of emulsified, unhydrolyzed lipids. Citrate utilization was observed as zones of clearing around colonies on calcium citrate medium [49]. The acidifying activity was assessed by inoculating (1% *w*/*v*) the strains in RSM and incubating them for 24 h at 30 °C. The values of pH were measured using a HI8520 pH meter (Pool Bioanalysis Italiana, PBI, Milan, Italy) after 6 and 24 h of incubation. The acidifying activity was expressed as the decrease in pH with respect to the value of non-inoculated control milk, as previously reported [43]. All experiments were made in duplicate.

### 2.4. Preparation of the Inoculums

Before manufacturing the cheese, each LAB strain was revitalized by incubation for 24 h at 30 °C in MRS broth. Individual colonies of each strain were transferred in RSM and incubated for 48 h at 30 °C to achieve a final concentration of about 10^7^ cfu/mL, which was confirmed with dilution and plating on MRS agar.

*L. monocytogenes* ATCC 7644 was seeded in NB and incubated for 18 h at 37 °C, then the revitalized cells were pelleted by centrifugation (Centrifuge 5804R, Eppendorf, Hamburg, Germany), for 10 min at 5900 g, washed twice, resuspended in buffered peptone water and diluted to achieve the inoculum concentration of 10^5^ cfu/mL, which was confirmed by enumeration on the selective medium Agar Listeria Ottaviani Agosti (ALOA, Microbiol).

### 2.5. Miniature Fresh Cheese Manufacture and “In Situ” Antilisterial Activity of LAB

Miniature fresh cheese was manufactured on a laboratory scale under aseptic conditions following the protocol reported in Supplementary Figure S1.

Pasteurized ewe’s milk (6.4% fat, *w*/*w*) kindly provided by a local dairy farm (Argiolas Formaggi, Dolianova, CA, USA) cooled to 35–37 °C was distributed in 500 mL aliquots in plastic containers and individually inoculated with each LAB strain (1%) at a concentration of about 10^7^ cfu/mL. *L. monocytogenes* (1%) was added to the milk at a concentration of about 10^5^ cfu/mL immediately after LAB inoculation. After mixing gently with a sterile glass rod, commercial liquid rennet was added to ensure coagulation. After 10 to 15 min at 37 °C, the curd was cut into 4 blocks using a sterile knife, the excess whey was discarded, and the containers were stored at 10 °C and 85% humidity for up to 7 days. The weight of the cheeses was about 100 g.

Miniature cheese manufacture was carried out on two different days, one for lactococci and one for lactobacilli strains, and two different kinds of cheese were made for each strain. In each experiment, control cheeses inoculated with *L. monocytogenes* only were included.

Cheeses were sampled in duplicate (two different kinds of cheese of each strain) for LAB and *L. monocytogenes* counts at the beginning (time 0) and at 1, 4 and 7 days of storage at 10 °C. Ten-gram aliquots of cheese were aseptically collected from each container, transferred to a sterile tube containing 90 mL of 2% (*w*/*v*) sodium-citrate sterile solution, homogenized in a Stomacher Lab Blender (PBI) for two minutes at normal speed, then serially diluted in sterile saline solution and plated onto MRS and ALOA agar plates for the enumeration of LAB and *L. monocytogenes*, respectively.

The pH value of the cheeses was determined using a HI8520 pH meter (PBI) at 1, 4 and 7 days of storage at 10 °C by homogenizing 10 g of cheese in 10 mL of distilled water.

### 2.6. Statistical Analysis

The results are depicted as mean with the standard deviation. Microbial counts were calculated as cfu per gram of sample and reported as log_10_ cfu/g. The data derived from microbiological analyses were evaluated using one-way analysis of variance (ANOVA) and Bonferroni’s multiple comparison test with GraphPad Prism Statistics software package version 5.00 (GraphPad Prism Software Inc., San Diego, CA, USA) in order to calculate the differences among the means. Significance was established at *p* < 0.05. Correlations between *L. monocytogenes* growth in the different treatments and the pH were calculated using Pearson’s correlation coefficient at 0.05 significant level.

## 3. Results and Discussion

Table 1 reports the evaluation of some technological activities in the LAB strains tested.

The majority of lactococci displayed proteolytic activity on casein agar after 24 h of incubation at 30 °C. The ability to hydrolyze casein was found to be absent in some *Lactiplantibacillus plantarum* and in the *Lactococcus lactis* strain 11FS16. This property, widely described in LAB strains, in particular in *L. lactis*, is linked to the formation of free peptides and amino acids that can play a role in the development of aromas in cheeses. None of the strains demonstrated lipolytic activity on tributyrin agar. The citrate-fermenting activity, used as an indicator of the production of aromatic substances, was found to be present in all lactobacilli strains and in the *L. lactis* strain 11FS16 that was therefore assigned to biovar *diacetylactis*. In regards to the acidifying activity, a technological parameter of particular importance, the strains analyzed were generally medium acidifiers, determining a lowering of the pH of the milk higher than one unit after 24 h of incubation at 30 °C, except for the strain *Lpb. plantarum* 4/16898, which caused a decrease in pH greater than 2 units. These results highlight a certain degree of variability in the technological characteristics examined not only between different species but also between strains belonging to the same species, indicating that these are properties mainly strain-related [50].

As shown in Table 2, most strains were able to antagonize *Listeria monocytogenes*, *Staphylococcus aureus* and *Escherichia coli* with clear inhibition zones of more than 4 mm in agar-spot plates. The lowest activity was detected against *E. coli*. A broad antagonistic activity of *Lactobacillus* towards different pathogenic microorganisms has been reported [51]; however, 27 strains of *Lpb. plantarum* isolated from cheese showed no activity against selected indicator pathogens [52]. Several in vitro studies have demonstrated that the antagonistic activity of *L. lactis* cultures against pathogens, including *L. monocytogenes*, was generally related to the production of bacteriocins [53,54]. As reported in the literature, LAB are capable of producing several different substances with antimicrobial power, including the major metabolic end products such as organic acids, hydrogen peroxide, ethanol and bacteriocins [55].

When tested against *L. monocytogenes* ATCC 7644 with the well diffusion technique according to Schillinger & Lücke [48], no residual antibacterial activity was observed in the *Lpb. plantarum* strains, while four strains of *L. lactis* exhibited inhibitory activity even after neutralization and catalase treatment of the supernatant, confirming their capability of producing antimicrobial compounds of proteinaceous nature, as shown in previous studies [37,43] (data not shown).

**Figure 1:**
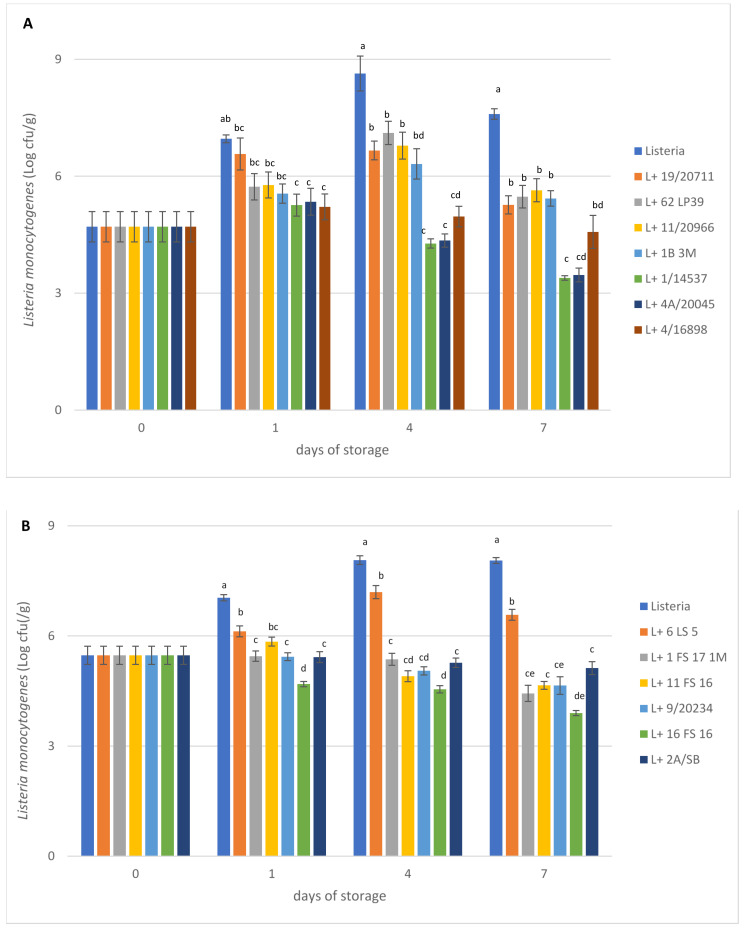
Effect of *Lactiplantibacillus* (**A**) and *Lactococcus* (**B**) strains on *Listeria monocytogenes* counts in experimental cheeses during storage (1, 4, 7 days) at 10 °C (means ± SD of two samples). Means corresponding to the same storage time with different letters are significantly different (*p* < 0.05).

The effect of the LAB strains on the growth of *L. monocytogenes* ATCC 7644 in miniature cheeses produced under laboratory conditions, evaluated by one-way analysis of variance performed among the different treatments, is presented in Figure 1. Growth of *L. monocytogenes* in control samples increased from about 10^5^ cfu/g to 10^7^ cfu/g within 24 h, reaching about 10^9^ cfu/g after 7 days of storage at 10 °C. In experimental cheeses, where *L. monocytogenes* was added together with the LAB strains, different trends in the growth were obtained and differences in the degree of inhibition were noted among the strains. In general, all LAB strains determined *L. monocytogenes* log reduction numbers with respect to the control ranging from 1.48 to 4.16 for lactococci and 1.96 to 4.21 for lactobacilli. Significantly lower (*p* < 0.05) *Listeria* counts were detected in cheeses produced with all LAB strains with respect to the control at 4 and 7 days of storage at 10 °C. Three strains (*L. lactis* 16FS16 and *Lpb. plantarum* 1/14537 and 4A/20045) were able to significantly reduce *L. monocytogenes* counts by about 4 log units compared to the control cheese and by 1.5 log units compared to the initial inoculum at 7 days of storage at 10 °C. Other lactococci strains (9/20234, 11FS16 and 1FS171M) showed good bacteriostatic activity, with a reduction of more than 3 log units compared to control. It is worth noting that the lactococci strains 11FS16, 9/20234 and 16FS16 were characterized in a previous study as producers of nisin A [43], but to the best of our knowledge, this is the first study reporting a strain of *L. lactis* biovar *diacetylactis* with inhibitory antilisterial potential in situ. The unexpectedly low antilisteria activity with *L. lactis* strain 6LS5, a nisin-Z producer [43], could indicate the presence of bacteriocin adsorption on the cell surface or on milk proteins or a low bacteriocin production in situ [56]. In the work by Dal Bello et al. [57], two *L. lactis* nisin-Z-producing strains also failed to inhibit *L. monocytogenes* in experimental cheese production, presumably because of the high inoculum (10^6^ cfu/mL) of the pathogen. Similar results were also obtained by various authors who observed the failure of the antagonistic activity of bacteriocin in cheese compared to the results obtained in vitro [24,58]. It has also been shown that some factors related to cheese composition (fat content, proteolytic degradation, sodium chloride concentration) can affect the effectiveness of bacteriocins [59].

Cell counts of the LAB strains were also recorded during the storage of cheese at 10 °C (Supplementary Figure S2). All strains grew well in cheese samples, reaching counts around 10^8^ and 10^9^ cfu/g for lactococci and lactobacilli, respectively, after 7 days.

Table 3 reports the evaluation of pH in control and experimental cheeses during storage. Values of pH generally decreased during storage and were reduced by approximately 2 units (1.5 to 2.5 units depending on the strain) at 7 days. The pH of cheeses was significantly influenced by both lactococci and lactobacilli strains during storage. A slight reduction in pH values during storage was also observed in control cheeses. Lactococci and lactobacilli are known to produce organic acids during cheese ripening, which are possibly accountable for the pH drop. It is known that the production of organic acid and decrease in pH values represent important factors in controlling the growth of *L. monocytogenes* [60]. In cheeses produced with lactococci strains 9/20234, 11FS16 and 16FS16 and with lactobacilli strains 1/14537 and 4A/20045, a significant correlation (*p* < 0.05) was observed between *Listeria* counts and pH values during storage. For the three lactococci strains, all nisin-producers, the antibacterial activity could be related to the combined production of both organic acids and antimicrobial metabolites, as already reported [61]. For the lactobacilli strains, the antilisterial activity could be ascribed to the production of organic acids, as we have recently shown in the case of strain 1/14537 [41]. In the work by Rogga et al. [34], the inactivation of *L. monocytogenes* in laboratory-scale Galotyri cheese was not related to the low pH of the cheeses produced with artisan-type inoculum because the cheeses produced with commercial strains supported the survival of the pathogen despite their even lower pH. Conversely, other studies have pointed out a lower activity of nisin according to pH of cheese: Henderson et al. [62] showed that nisin is more effective when cheese is made at pH 6–6.5, and Samelis et al. [59] found that in situ antilisterial effects of nisin are limited in cheese with a low pH (5.5–5.8).

Although in situ antilisterial activity of LAB strains has been evaluated in several studies, comparing the results is often difficult due to the different strains, type of antagonistic compounds, differences in cheese type, ripening period and storage temperature.

A reduction of 2–3 log units of *Listeria* counts in cheese were recorded after 7 days of ripening using bacteriocinogenic *L. lactis* [63]. In the study by Kondrotiene et al. [54], three nisin-producing *L. lactis* strains showed significantly reduced *Listeria* numbers by 2 log units in fresh cheese during 7 days storage at 4 °C. On the other hand, in the work by Samelis et al. [59], the application of a commercial starter culture with a nisin-A-producing *L. lactis* strain failed to determine a major (>3 to 5 log units) in situ reduction in *Listeria* counts in Greek Graviera cheese due to weak or undetectable nisin activity. The addition of *L. plantarum* as an adjunct starter was successful in controlling *L. monocytogenes* during storage in ultra-filtered white cheese [64] and LAB, including two *L. plantarum* group strains, isolated from traditional cheeses produced in Calabria were effective both in vitro and in soft cheese in lowering *Listeria* counts from 0.5 to 1 log cfu/g [65], while in Minas Frescal cheese, the *L. plantarum* ALC01 strain did not significantly affect listerial counts [66].

## 4. Conclusions

Among the microorganisms of technological interest, LAB are considered one of the most relevant microbial groups for their use as starter/adjunct cultures in the production of numerous fermented foods. In addition to conferring typical aromas and flavors, they exert an important protective effect, so the selection and use of indigenous lactic cultures to inhibit or reduce pathogens development and improve the quality of cheeses represents an alternative strategy to the various technological operations and thermal, physical, physico-chemical and chemical treatments. The antimicrobial action of LAB may be expressed through the competition for nutritional elements and the production of metabolic compounds, including organic acids, bacteriocins and bacteriocin-like substances. In our study, five strains (*L. lactis* 16FS16-9/20234-11FS16 and *Lpb. plantarum* 1/14537-4A/20045) could be considered good candidates for use as protective cultures while manufacturing fresh cheese. Our results suggest that the single and combined action of the acidifying power and the production of bacteriocin in these strains was capable of controlling and/or reducing the growth of *Listeria monocytogenes*. Considering their technological characteristics, they could be used as starter/adjunct cultures to improve the safety of the products, perhaps in association with other antimicrobial hurdles, as suggested [58].

The results obtained at the laboratory scale are promising; however, further studies are needed on the evaluation of these strains according to safety criteria and on the determination of metabolites and antimicrobial compounds produced by the selected *L. lactis* and *Lpb. plantarum* strains in order to evaluate their efficacy in controlling the growth of *L. monocytogenes* during cheese manufactured at factory scale.

Furthermore, these autochthonous strains, used as biopreservative agents, could represent a useful tool for the protection of the microbial biodiversity present in the Sardinian territory.

## Figures and Tables

**Table 1:** Technological characteristics of the autochthonous strains.

Strains	Casein Hydrolysis	Lipolytic Activity §	Citrate Utilization	Acidifying Activity *	
				ΔpH (6 h)	ΔpH (24 h)
*Lactococcus lactis*					
16FS16	+	-	-	0.91 ± 0.05	1.84 ± 0.13
11FS16	-	-	+	0.73 ± 0.11	1.22 ± 0.10
6LS5	+	-	-	0.71 ± 0.08	1.44 ± 0.07
1FS171M	+	-	-	0.64 ± 0.08	1.24 ± 0.15
2A/SB	+	-	-	0.41 ± 0.06	1.90 ± 0.03
9/20234	+	-	-	0.57 ± 0.09	1.39 ± 0.12
*Lactiplantibacillus plantarum*					
62LP39b	+	-	+	0.66 ± 0.10	1.14 ± 0.14
11/20966	+	-	+	0.67 ± 0.09	1.29 ± 0.07
4A/20045	-	-	+	0.50 ± 0.11	1.14 ± 0.13
19/20711	+	-	+	0.60 ± 0.03	1.08 ± 0.12
1B3M	-	-	+	0.63 ± 0.12	1.15 ± 0.16
4/16898	+	-	+	1.09 ± 0.09	2.22 ± 0.11
1/14537	-	-	+	1.02 ± 0.05	1.76 ± 0.09

§ on Tributyrin agar. * Values presented are means ± SD of two replicate evaluations for each strain.+ positive reaction, - negative reaction.

**Table 2:** Antibacterial activity of autochthonous strains determined by the agar spot test.

Strains	*S. aureus* ATCC 25923	*E. coli* ATCC 25922	*L. monocytogenes* ATCC 7644
*Lactococcus lactis*			
16FS16	+	-	+
11FS16	+	-	+
6LS5	+	-	+
1FS171M	+	+	+
2A/SB	+	+	+
9/20234	+	-	+
*Lactiplantibacillus plantarum*			
62LP39b	+	+	+
11/20966	+	+	+
4A/20045	+	+	+
19/20711	+	+	+
1B3M	+	+	+
4/16898	+	+	+
1/14537	+	+	+

+ positive reaction (inhibition zone ≥ 4 mm). - negative reaction (inhibition zone < 4 mm).

**Table 3:** pH of cheeses measured at 1, 4 and 7 days of storage at 10 °C after inoculation with *L. monocytogenes* ATCC 7644 and each LAB strain (means ± SD of two replicates for each sample).

	Cheese pH Days of Storage at 10 °C			
Cheese Made with Strain		1	4	7
*Lactococcus lactis*	pH of milk: 6.57			
	Control *	6.42 ± 0.03 ^a^	5.75 ± 0.08 ^a^	5.37 ± 0.14 ^a^
	16FS16	5.22 ± 0.08 ^b^	4.59 ± 0.09 ^b^	4.50 ± 0.12 ^b^
	11FS16	5.48 ± 0.04 ^bce^	4.65 ± 0.02 ^b^	4.51 ± 0.07 ^b^
	6LS5	5.65 ± 0.04 ^c^	4.68 ± 0.01 ^c^	4.28 ± 0.04 ^b^
	1FS17 1M	5.12 ± 0.06 ^b^	4.55 ± 0.07 ^b^	4.29 ± 0.09 ^b^
	2A/SB	4.33 ± 0.16 ^d^	4.10 ± 0.08 ^d^	4.08 ± 0.11 ^b^
	9/20234	5.74 ± 0.08 ^e^	5.28 ± 0.04 ^a^	5.06 ± 0.06 ^a^
*Lactiplantibacillus plantarum*	pH of milk: 6.68			
	Control *	6.25 ± 0.37 ^ab^	5.84 ± 0.15 ^a^	5.47 ± 0.10^a^
	62LP39b	6.28 ± 0.04 ^a^	4.44 ± 0.04 ^b^	4.23 ± 0.09 ^b^
	11/20966	6.32 ± 0.12 ^a^	4.61 ± 0.05 ^bc^	4.22 ± 0.04 ^b^
	4A/20045	6.04 ± 0.08 ^ab^	4.88 ± 0.10 ^cd^	4.36 ± 0.03 ^b^
	19/20711	6.36 ± 0.04 ^a^	4.41 ± 0.04 ^b^	4.27 ± 0.08 ^b^
	1B 3M	6.37 ± 0.05 ^a^	4.49 ± 0.02 ^bc^	4.26 ± 0.06 ^b^
	4/16898	5.44 ± 0.23 ^b^	4.64 ± 0.09 ^bcd^	4.29 ± 0.02 ^b^
	1/14537	5.97 ± 0.22 ^ab^	5.03 ± 0.13 ^d^	4.61 ± 0.04 ^c^

Means corresponding to the same storage time with different letters are significantly different (*p* < 0.05). * Cheese inoculated with *Listeria monocytogenes* only.

## Data Availability

The mean ± standard deviation of microbial data used to support the findings of this study are included in the article. The raw data are available on reasonable request from the corresponding author.

## Supplementary Materials

The following supporting information can be downloaded at: https://www.mdpi.com/article/10.3390/foods11050715/s1, Figure S1: Flow diagram of the cheese manufacturing procedure; Table S1: In vitro characteristics related to technological and antimicrobial properties of the microbial strains used in the study; Figure S2: title: Evolution of Lactococci (A) or Lactobacilli (B) counts in experimental cheeses during storage (0, 1, 4, 7 days) at 10 °C (means ± SD of two samples).
