# Supplementary material for: Inhibitory Effect of Lactiplantibacillus plantarum and Lactococcus lactis Autochtonous Strains against Listeria monocytogenes in a Laboratory Cheese Model
Source: Foods. 2022 Feb 28;11(5):715. doi: 10.3390/foods11050715 (PMC8909851; doi:10.3390/foods11050715)
Supplement: Supplementary file 1 [file foods-11-00715-s001.zip › foods-1600023-supplementary.pdf]

SUPPLEMENTARY MATERIAL

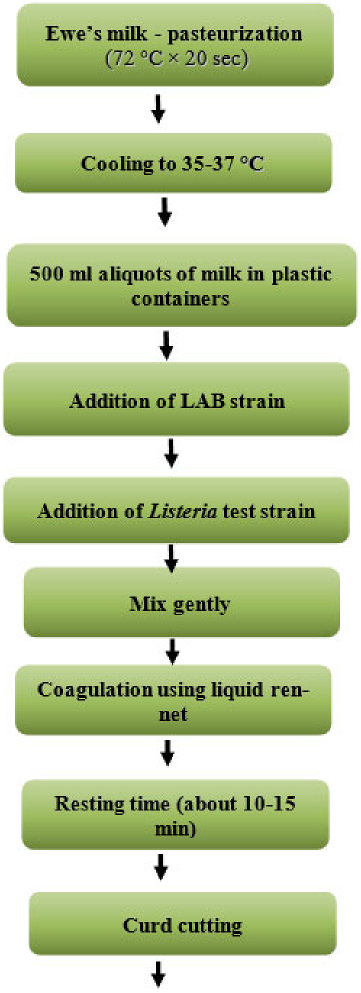

Figure S1. Flow diagram of the cheese manufacturing procedure.

Table S1. -In vitro characteristics related to technological and antimicrobial properties of the microbial strains used in the study.

Microbial strains	Isolated from	MBDS code #	Technological properties	Antimicrobial properties	Reference
L. lactis 16FS16	Sheep cheese	UNICA B39	• 6.5% NaCl +<br>• *B*-galactosidase +<br>• Nisin A producer	• Antimicrobial activity<br>• Antilisterial activity in vitro	Cosentino et al., 2012 [37]<br>Pisano et al., 2015 [43]<br>Siroli et al., 2019 [44]<br>Bukvicki et al., 2020 [45]
L. lactis 11FS16	Sheep cheese	UNICA B40	• 6.5% NaCl +<br>• *B*-galactosidase +<br>• Nisin A producer	• Antimicrobial activity<br>• Antilisterial activity in vitro	Cosentino et al., 2012 [37]<br>Pisano et al., 2015 [43]<br>Siroli et al., 2019 [44]<br>Bukvicki et al., 2020 [45]

L. lactis 6LS5	Raw sheep milk	UNICA B46	• 6.5% NaCl +<br>• *B*-galactosidase +<br>• Nisin Z producer	• Antimicrobial activity<br>• Antilisterial activity in vitro	Cosentino et al., 2012 [37]<br>Pisano et al., 2015 [43]<br>Siroli et al., 2019 [44]<br>Bukvicki et al., 2020 [45]
L. lactis 1FS171M	Sheep cheese	UNICA B108	• Milk coagulation +<br>• 6.5% NaCl +	• Antimicrobial activity	Cosentino et al., 2002 [46]<br>Master thesis (unpublished)
L. lactis 2A/SB	Sheep cheese whey	UNICA B56	• Milk coagulation +	• Antimicrobial activity	Cosentino et al., 2002 [46]<br>Master thesis (unpublished)
L. lactis 9/20234	Raw sheep milk	UNICA B47	• 6.5% NaCl +<br>• *B*-galactosidase +<br>• Nisin A producer	• Antimicrobial activity<br>• Antilisterial activity in vitro	Cosentino et al., 2012 [37]<br>Pisano et al., 2015 [43]<br>Siroli et al., 2019 [44]<br>Bukvicki et al., 2020 [45]
Lpb. plantarum 62LP39b	Raw sheep milk	UNICA B28	• Milk coagulation +	• Antibacterial activity<br>• Antifungal activity	Pisano et al., 2008 [47] (*L. plantarum* DBS273 renamed as *L. plantarum* 62LP39B)
Lpb. plantarum 11/20966	Raw sheep milk	UNICA B26	• Milk coagulation +<br>• *B*-galactosidase +	• Antibacterial activity<br>• Antifungal activity	Cosentino et al., 2002 [46]<br>Pisano et al., 2014 [39]<br>Cosentino et al., 2018 [3]
Lpb. plantarum 4A/20045	Raw sheep milk	UNICA B29	• Milk coagulation +	• Antifungal activity	Cosentino et al., 2002 [46]<br>Cosentino et al, 2018 [3]
Lpb. plantarum 19/20711	Raw sheep milk	UNICA B25	• Milk coagulation +<br>• *B*-galactosidase +	• Antibacterial activity<br>• Antifungal activity	Cosentino et al., 2002 [46]<br>Pisano et al., 2014 [39]<br>Cosentino et al., 2018 [3]
Lpb. plantarum 1B3M	Sheep cheese	UNICA B30	• Milk coagulation +	• Antifungal activity	Cosentino et al., 2002 [46]<br>Cosentino et al, 2018 [3]
Lpb. plantarum 4/16898	Raw sheep milk	UNICA B27	• Milk coagulation +<br>• *B*-galactosidase +	• Antibacterial activity<br>• Antifungal activity	Cosentino et al., 2002 [46]<br>Pisano et al., 2014 [39]<br>Cosentino et al., 2018 [3]
Lpb. plantarum 1/14537	Raw sheep milk	UNICA B32	• Milk coagulation +	• Antifungal activity	Cosentino et al., 2002 [46]<br>Cosentino et al, 2018 [3]

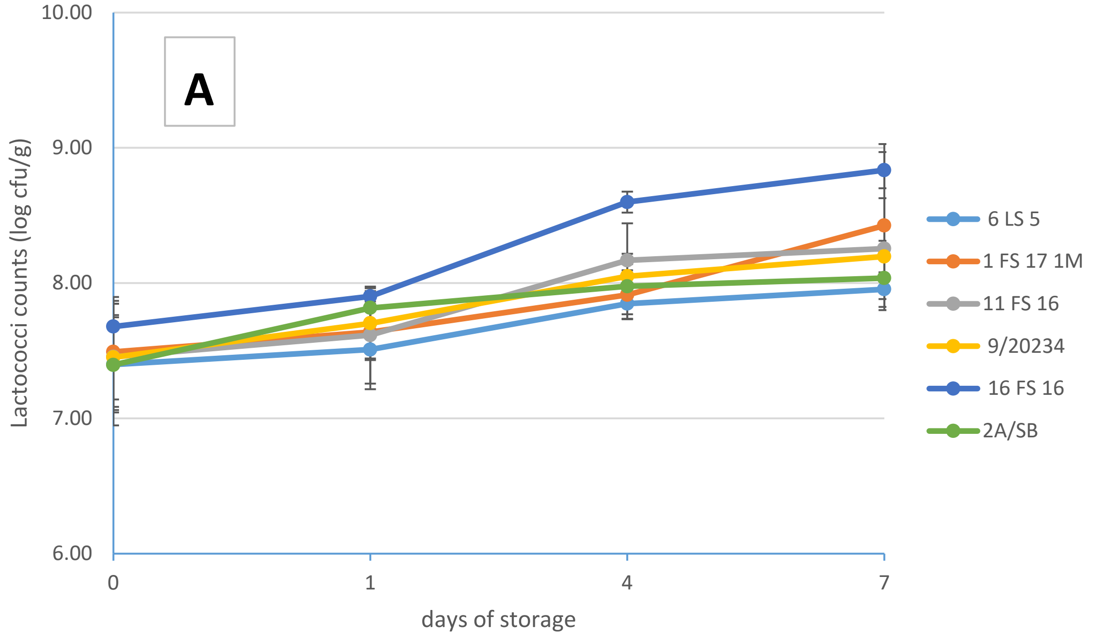

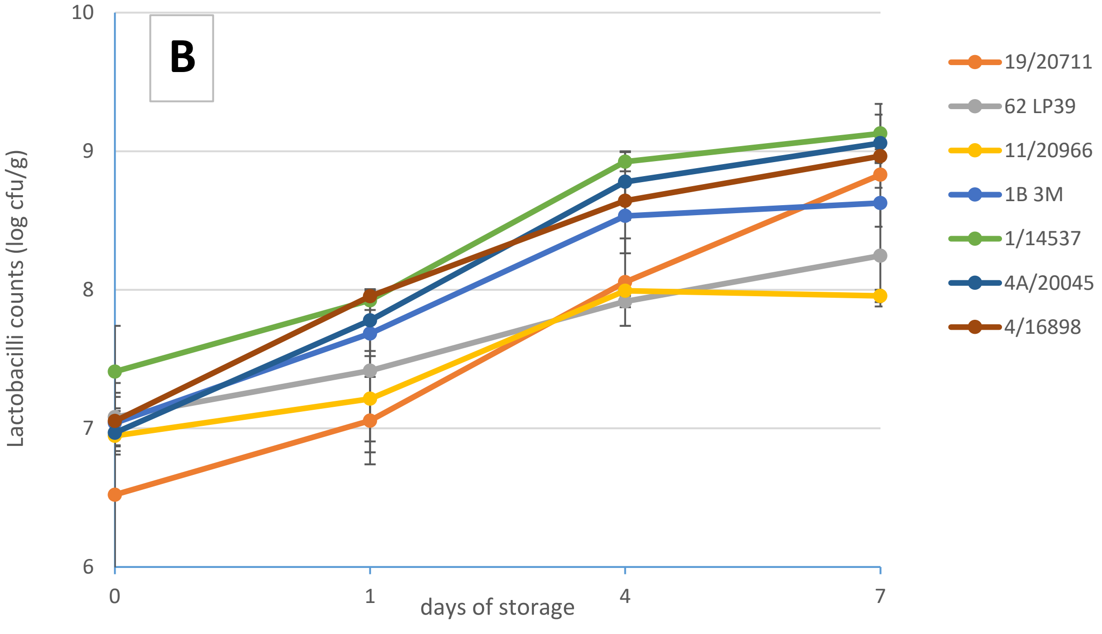

Figure S2. Evolution of Lactococci (**A**) or Lactobacilli (**B**) counts in experimental cheeses during storage (0, 1, 4, 7 days) at 10°C (means ± SD of two sample
